# Developing a collegiate recovery program from the ground up: identifying priorities, promoting collaboration, assessing needs, and offering recommendations

**DOI:** 10.1186/s13722-025-00630-6

**Published:** 2025-12-24

**Authors:** Mary B. Tabit

**Affiliations:** Department of Psychology, University of Scranton, Scranton, 18510-4596 PA USA

**Keywords:** Collegiate recovery program, CRP, Program development, Substance use, Addiction, Recovery, College student health

## Abstract

**Background:**

Addiction in its many forms is a pervasive public health challenge that afflicts millions of people. With adolescence identified as the at-risk period for developing substance use disorders (SUDs), and young adults in the U.S. steadily demonstrating the highest SUD prevalence rates among individuals aged 12 and older, providing supports to college-aged youth in recovery or recovery curious is critical to promoting positive social, health, and academic outcomes.

**Case presentation:**

Collegiate Recovery Programs (CRP) are proliferating across the nation as a valuable campus resource for students in recovery from SUDs and process addictions. The purpose of this article is to provide an overview and first-person account of developing a CRP at a 4-year institution of higher education in the U.S., over the course of one year, through the lens of a CRP co-director.

**Conclusions:**

Program development, particularly in a space like CRPs where no one “gold standard” model exists, and individualizing programming to campus culture is key, is a time and resource-intensive process that evolves over time. Materials created to support program development are included and recommendations emerging from this experience are discussed.

## Background

Despite college attendance serving as a protective factor for decades, recent data identifies substance use as one of the most pervasive health problems facing college campuses across the United States [1]. Substance use and disorder rates and trends, combined with risky use indicators among young adults generally, and college students in particular, provide critical perspective into this public health concern [2]. Recent national level survey data reveals that in 2024 “young adults had historically high prevalence levels” of cannabis use, nicotine vaping, and hallucinogens/psychedelics [2]. In fact, the highest prevalence rate for daily cannabis use ever recorded among college women was observed in 2024 at 6.8% [2]. Further, National College Health Assessment (NCHA*) survey data reveals that 15.3% of college students are engaging in moderate risk use of tobacco or nicotine delivery products, 17.4% are engaging in moderate risk use of cannabis (nonmedical use), and 9.1% are engaging in moderate risk use of alcohol [3]. Finally, past year substance use disorder (SUD) rates in the U.S. have remained highest among 18 to 25 year olds since 2021, with approximately 26% of 18–25 year olds endorsing a past year SUD in 2024 [4]. Researchers have identified several potential contributors to these rates, including the history of substance use being woven into the tapestry of college tradition, normative acceptance of substance use among peers, and lower perceived risk of harm [1, 5, 6]. Dismantling misconceptions integral to these modes of thinking, providing harm reduction, and making recovery supports accessible on all college campuses is paramount.

College student alcohol and other drug (AOD) use is correlated with myriad negative health, social, and academic outcomes [1] including reduced physical and mental health, engagement in risky behavior, and school drop-out [3, 7]. Further, the harms associated with college student substance use extend beyond the individual user, as evidenced by research demonstrating that over half of U.S. college sophomore and junior students experienced at least one alcohol-related harm resulting from *another* person’s drinking (e.g., unwanted sexual contact, physical or psychological distress, disruptions in sleep and studying) [8, 9].

Encouragingly, recent estimates indicate that among the 2.8 million young adults aged 18–25 in the U.S. who endorse ever having a problem with alcohol or other drug use (AOD) in their lifetime, over 65% identify as being in recovery or having recovered [10]. Further, NCHA survey data indicates that, in 2025, 2.3% of college students identify as being in recovery from AOD use [3]. *Recovery* has been defined as “a process of change through which individuals improve their health and wellness, live self-directed lives, and strive to reach their full potential” [11]. Sustaining recovery while pursuing an undergraduate degree is not without its challenges—in fact, some experts have referred to the college campus as a “recovery hostile environment” [12]. Peer binge drinking, university-assigned housing, stigmatization, and media influence have all been identified as posing risks to recovery status among college students [13].

Myriad approaches have been implemented in higher eduation to reduce risky AOD use. These range from *prevention* (e.g., abstinence policies and university-imposed sanctions; campus-wide educational efforts; universal screening and feedback) to *early intervention* (e.g., connection to on and off campus resources) to *treatment* (e.g., peer-led support interventions; individual and group counseling; harm reduction activities), ending with or continuing with *recovery*, including mutual support groups (e.g., 12-step meetings conducted on campus) and Collegiate Recovery Communities and Programs (CRC/Ps).

Broadly defined, CRPs are institutionally sanctioned spaces for students in higher education who identify as being in recovery from AODs and process addictions, designed to promote health and academic success [14]. CRPs are ideally grounded in the unique needs of each campus community, and therefore, no set formula for CRP programming, policies, and administration exists to date [12, 15]. Program components typically include peer to peer mutual support, social activities designed to foster connection and substance-free fun, and tools aimed at promoting academic and social success [12]. Best practices emphasize inclusive definitions of recovery that bolser a strong sense of connectedness; a dedicated space for CRP members to gather; a robust range of programmatic elements to support sustained recovery; and collaborative relationships among on and off-campus offices and organizations [12, 15].

Several models have been proposed to explain how CRP involvement is thought to promote long-term recovery. The *Recovery Capital Framework* [16] posits that, as students in recovery are provided with, and utilize resources, at the cultural, social, and environmental levels, this builds one’s *recovery capital* [5, 16, 17]. Building capital in one domain tends to have a positive, synergistic effect on other domains, which promote positive change and outcomes among college students in recovery [16]. Adding to this, the *Socioecological Model for CRPs* [17] situates students within the broader context of which they are a part, to demonstrate the ways in which different factors and systems interact and bidirectionally influence one another to facilitate or hinder academic and recovery-related success (i.e., individual, interpersonal, organizational, and policy). Taken together, these theories offer insights into the change process thought to be associated with CRP participation [16, 17].

The number of CRPs on college campuses has grown substantially in recent years. The first CRP was developed at Brown University in the late 1970s [18]. In 2010 “just a handful” of CRPs existed [19], whereas in 2025, there are 183 known CRPs [20]. To ensure programmatic quality, while maintaining responsiveness to student needs and campus culture, the Association of Recovery in Higher Education (ARHE) launched a CRP review and accreditation process in 2023 [21]. Of the 183 existing CRPs, the ARHE has reviewed 11 programs to date [21]. Undoubtedly, this work will lend itself to more extensive empirical review of the essential elements of CRPs and how to leverage these to further facilitate positive outcomes.

In the interim, Vest and colleagues [13] conducted a PRISMA-guided scoping review to answer the question *what is known from the existing literature about SUD recovery programming in higher education?* Findings indicate that students in CRPs are more likely to graduate from college, exhibit higher retention rates, and have higher GPAs than other students at the same respective institutions [13]. Additionally, students who have access to CRP programming on their campus as well as CRP alumni are less likely to return to AOD use compared to students who did not have CRP programming available [13, 22–26], and receipt of abstinence support via CRPs is correlated with reduced cravings to use substances among student participants [13, 27–29]. Qualitative research included in the Vest et al. review identified elements of CRPs that participants have found important, including an accessible student drop-in center; availability of mutual support programming on campus; strong peer and community support within the CRP; focus on diversity; and supports to promote academic success, health, and wellness (e.g. [13, 30–35]).

Three stigma-related studies were included in Vest and colleagues [13] review. The first study demonstrated that students, staff, and faculty who were offered CRP recovery ally trainings were more likely to utilize sensitive, inclusive verbiage; talk with others about their campus CRP; and endorse less stigma towards people in recovery. Another study showed increased empathy for students in recovery among student allies, and the third study utilized pictures to demonstrate CRP participants’ experiences of stigma [13, 36–38].

Given the positive outcomes associated with CRPs and their flexibility in responding to student needs and institutional culture, starting a CRP can feel equal parts invigorating and overwhelming: invigorating in making a positive impact on a young person’s collegiate career and beyond through the provision of community and support, and overwhelming in that each decision carries substantive implications in terms of student experience, program success, and sustainability. Building upon existing resources and empirical literature, the purpose of this article is to provide an overview and first-person account of the steps taken to develop a CRP at a 4-year predominantly white institution of higher education in the United States. The account occurs over the course of one year, through the lens of a CRP co-director. We start with the document *Getting Started: What You Need to Know About Building a CRP* (see [12]) and trace the specific steps taken by our team throughout the development process. We furnish examples of the materials created to support action steps, with the goal of streamlining the CRP development and implementation process, including identification of students in recovery and their allies, for other campuses looking to provide this evidence-based resource to their student population.

## Case presentation

### CRP development: Identifying and defining focal areas for our campus

In collaboration with my co-director—a substance use disorder subject matter expert, licensed clinican, and tenured faculty member in a leadership role with extensive knowledge of the institution’s history—our program development began with an open-ended question: How can we best support our students in recovery? We reflected on our anecdotal experiences with the students in our classrooms, their parents and caregivers, health professional trainees, local community organizations, and fellow colleagues, identifying in the process a running list of what we perceived to be current student needs; administrative, institutional, and community resources, supports, and potential barriers; previous campus efforts to support students in active addiction and recovery and potential partnerships; short and long term goals for developing the CRP; and a candid assessment of our personal resources given our life and work roles and responsibilities to prioritize areas of focus (e.g., hosting CRP activities when one of us is already on campus should an urgent situation arise; setting reasonable expectations in terms of number of weekly CRP activities). This discussion resulted in a strategic framework for year 1 of CRP development, which included the following components:

1. Organize a CRP core development team, comprised of students and faculty, to guide the creation, implementation, and ongoing evaluation of the CRP.

2. Assemble a CRP task force comprised of students, faculty, staff, and administrators to better understand CRP campus readiness (i.e., student needs, anticipated barriers, potential resources) and identify potential collaborations across campus.

3. Conduct a landscape review of existing campus and community resources for students in recovery and/or active addiction.

4. Formally assess student recovery needs and interests to inform CRP programmatic elements.

5. Develop and launch at least one (1) weekly CRP activity on campus (e.g., peer support group) within eight (8) months.

6. Apply to at least one (1) professional conference or journal outlet to contribute to the scholarly literature on CRPs.

Each of these components, including the tasks undertaken in each domain, and links to materials we utilized to organize these efforts, are described.

#### Component 1: Organize a CRP core development team, comprised of students and faculty, to guide the creation, implementation, and ongoing evaluation of the CRP

Program development experience combined with our grant-provided technical assistance team informed the general skills and characteristics we wanted represented within our core CRP development team (i.e., project management, event planning, social media/marketing content development, data analysis skills, passion for working within the SUDs/recovery community). Students were recruited for the core development team via direct email communication/word of mouth and underwent interviews with the co-director(s) to better understand interest and relevant experience. Our core team ultimately consisted of two graduate student research assistants (RAs), one faculty member with SME in research methods and analysis, and two co-directors with SME in public health, program development, and SUDs.

#### Component 2: Assemble a CRP task force, comprised of students, faculty, staff, and administrators, to better understand CRP campus readiness (i.e., student needs, anticipated barriers, potential resources) and identify potential collaborations across campus.

It was important to us to assemble a diverse mix of students, faculty, staff, and administration in the planning, ongoing assessment, and implementation of the CRP. We were particularly interested in identifying individuals with lived experience (i.e., recovery identities, allies) who wanted to take on leadership roles, as we believed this would promote program visibility and sustainability. Further, we sought diverse representation in terms of roles and responsibilities within the university to encourage robust perspective-taking, critical analysis, and problem solving, while keeping the ask (i.e., time and resources) as low as possible to support consistent engagement. We envisioned and encouraged shared power within the task force by having transparent conversations about the bounds of our knowledge, making known our desire to engage in bidirectional support, and expressing genuine interest in learning from our students and colleagues. With this in mind, we asked potential task force members to commit to attending a 1-hour meeting each month to discuss recovery needs on campus and identify potential collaborations.

**Fig. 1:**
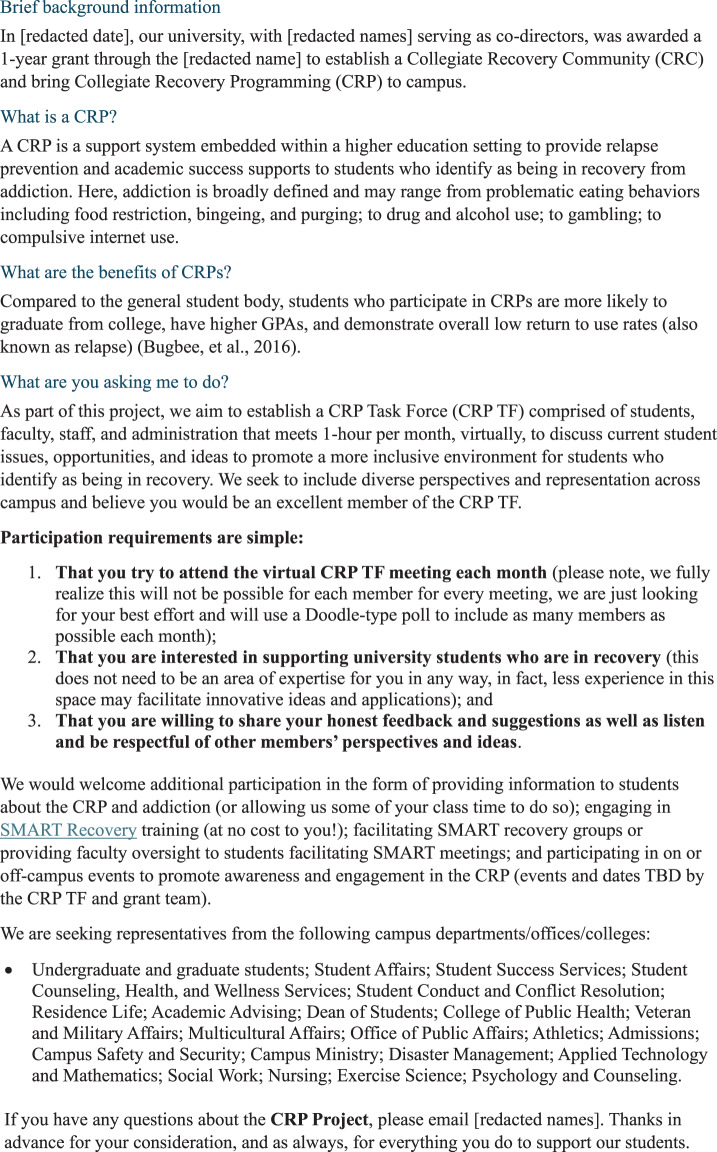
Collegiate recovery program university task force: invitation to participate

In terms of initial outreach, we created a table with each department identified in the *Getting Started* guide from ARHE and IBX [12]. We eliminated departments/offices that did not exist on our campus and added departments unique to our campus from which we wanted representation. We then identified the person within each of these departments/offices that we thought might be the best fit for our efforts, given content in their bios, previous experience, or current position. Figure 1 provides a sample letter for introducing and inviting members to join such a task force.

**Fig. 2:**
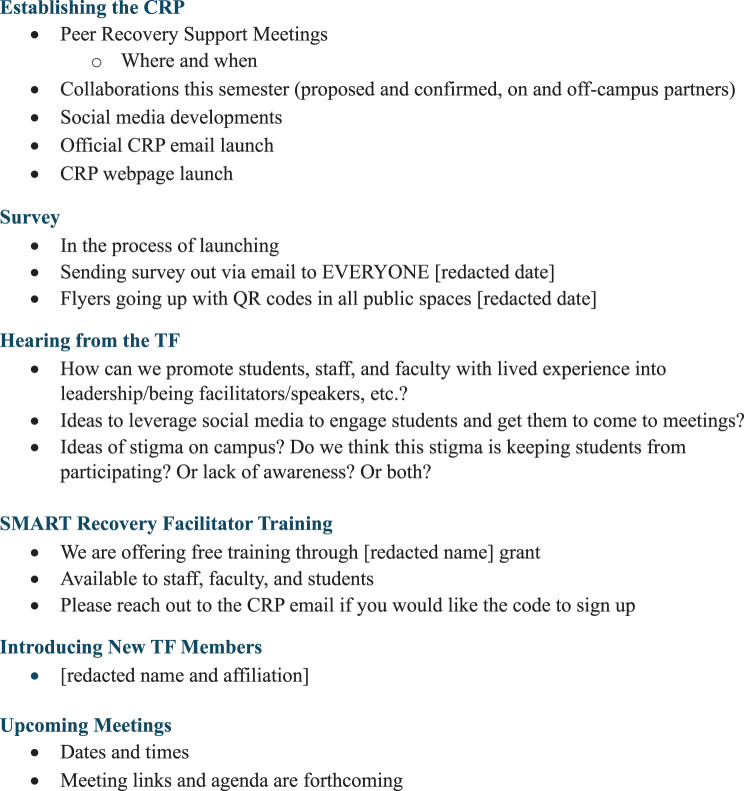
CRP task force: meeting agenda

Our recruitment efforts were successful; all but one outreached office agreed to join the task force, and to the author’s knowledge, only one individual left the task force after joining due to competing responsibilities. Monthly task force meetings were organized and facilitated by the CRP co-directors. Agenda items focused on topics the core team was grappling with (e.g., when to hold student programming; generating student interest; how to best collaborate with internal and external stakeholders), collating resources, and designing marketing strategies to make students aware of the CRP and how to get involved. Figure 2 offers a sample Task Force agenda.

In exploring our campus readiness (see 8, p. 5), we discovered that no previous recovery-specific efforts had been undertaken on our campus. Hence, we focused on previous and current campus-wide prevention and intervention efforts (whether these emerged through conduct-related, concerned other, or self referrals). This review was fluid; it started with the CRP co-directors and expanded to include current students, administrators, and colleagues on our task force who collectively represented diverse health and academic offices. Our questions centered on *current and previous substance use support-related endeavors* (e.g., What supports are available? What has been tried in the past? What has/not worked and what is your sense as to why?); *perceived gaps* (e.g., What is something you wish was available on campus to support students in recovery given your current role that is not? What student needs exist that are/are not being well met? What ideas do you have to best serve these needs? What would you like to know about student substance use/recovery to better inform your work?); *lessons learned from experience and suggestions for our efforts* (e.g., What challenges have you faced? How did you address these and what was the outcome? What would you do differently and why? What has been most helpful to you in your work?); *organizing a team* (e.g., individuals on campus involved in previous efforts and/or passion for working with students who may want to join our CRP core development team or task force); and *existing strengths* and how we could leverage these to realize our goals.

Insights gleaned from these conversations, including how and why university AOD policies and implementation procedures evolved over time; substance use trends among students; identified needs across campus offices to best support students in recovery or curious about recovery; and recommendations for individuals and organizations to connect with to support our efforts helped the co-directors solidify and prioritize our initial focus areas.

#### Component 3: Conduct a landscape review of existing campus and community resources for students in recovery and/or active addiction

To facilitate a deeper sense of community and connection, and to avoid duplication of services, we compiled a list of existing on-campus (e.g., counseling center, student health center, academic advising, career development) and off-campus resources (e.g., local treatment centers, grassroots organizations, harm reduction coalitions, national support organizations, mutual support groups). This was accomplished primarily through internet searches and discussions with students and colleagues. We outreached (cold-called or emailed) local organizations to explore how we could best support the broader recovery community and include their teams in our efforts. This led to speaking engagements (e.g., classroom event by a licensed clinician in recovery); two large on-campus events featuring a panel discussion of subject matter experts (SMEs); a campus-wide resource drive benefitting a local harm reduction coalition; a volunteer event at a harm reduction site; insights regarding existing community resources, trends, and needs; and new partnerships.

Our team also reviewed the mission, values, standard operating procedures (SOPs), and programming offered at other institutions’ CRPs. This consisted of looking at public webpages on university websites for event calendars, descriptions of CRP activities, program philosophy statements, and guiding principles. This review helped to provide a sense of how institutions were adapting the CRP model to meet the needs of their campus community, potential partnerships to explore to further support our efforts and expand our visibility, ways to promote sensitivity and inclusivity (e.g., how to subtly identify activities as being recovery-friendly by using a specific graphic on event fliers), common programmatic elements being offered and creative ideas for social events and activities, and the clear importance of using social media and university platforms to build peer connection and recovery community.

#### Component 4: Formally assess student recovery needs and interests to inform CRP programmatic elements

To promote student-centeredness, we aimed to identify student needs in real time, track these needs over time, and build/modify CRP programming accordingly. To do so, we requested previously collected university-level data to get a general sense of substance use among students and identify areas to further assess. This data proved difficult to access and/or interpret clearly for myriad reasons (e.g., questions were modified from validated assessment tools, staff who conducted original data collection were no longer with the university to explain these changes). We pivoted to developing and disseminating our own survey items to capture substance use and recovery culture on campus, student perceptions related to peer substance use, and student interests related to CRP programming. Survey item development consisted of several steps: first, the co-directors consulted with two CRP SMEs who provided technical assistance in the form of sharing their respective student needs-related survey tools to use as a starting point and providing iterative feedback on multiple drafts of our survey. Key campus stakeholders including colleagues in admissions, academic advising, student life, residence life, counseling and student wellness, and students on our core development team provided us with insights and feedback based on their experience and review. After multiple rounds of revisions, we ultimately landed on assessing these domains:

1. The frequency and type of substances being used.
2. The degree to which students perceive AOD use on campus is greater, less than, or equal to AOD use on other college campuses.
3. Identified needs among students in, attempting, or curious about recovery, including programmatic interests (e.g., peer support, academic resources, social activities).
4. Student availability and preferences regarding meeting times and contact method to promote engagement and support launch.

The survey was deployed using an online survey platform. Students were made aware of the survey through posted fliers on campus, QR codes on screens in common areas, and direct email communications.

**Fig. 3:**
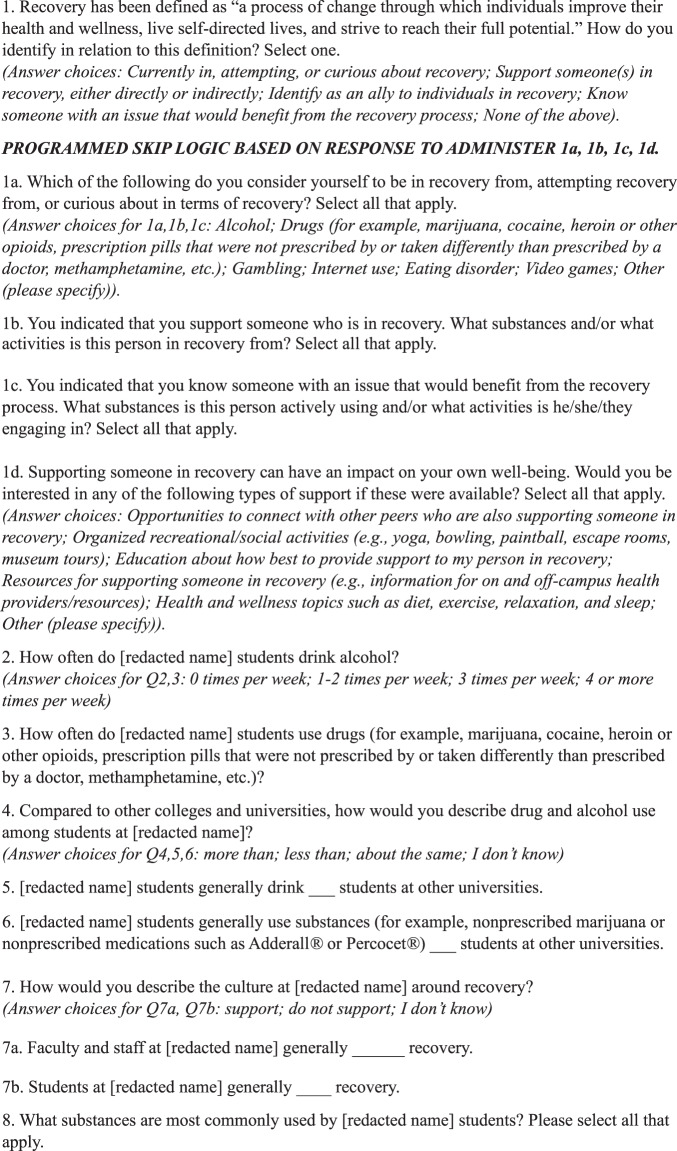

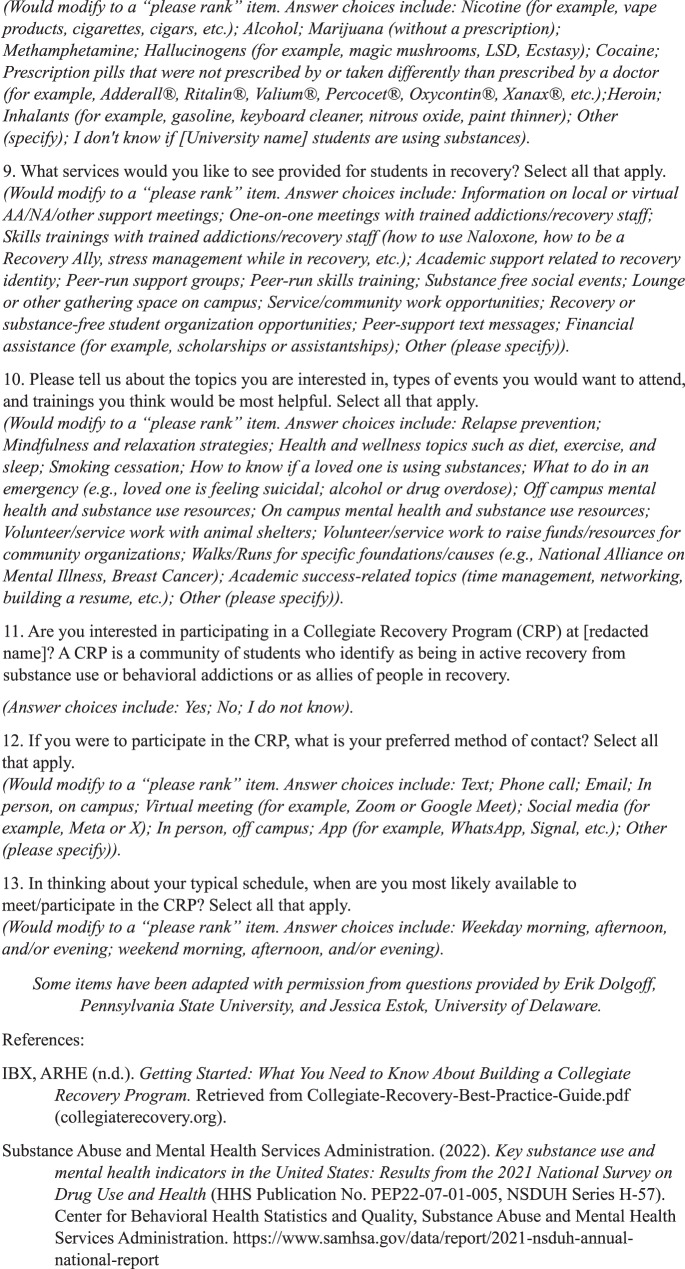
CRP Student needs and interests assessment items

Due to a low response rate resulting in a small, nonrepresentative sample, and unforeseen challenges with interpreting the data, survey results are not being reported here. For example, we employed *select all that apply* response options for several survey items. This provided breadth of information (e.g., what substances students are using, CRP programming topics of interest) but lacked critical depth for our purposes (e.g., made it impossible to determine which substances are most problematic on campus for us to target, or the programming options in which students are most interested to support prioritization). Figure 3 provides an abbreviated version of the survey with recommendations to enhance information depth and therefore CRP development.

#### Component 5: Develop and launch at least one weekly CRP program on campus (e.g., peer support group) within eight months

To inform initial programming, members of the core CRP development team reviewed the research base on effective CRP elements (e.g. [12–14, 16, 17, 31, 39]) and public information around CRP program offerings on collegiate websites (e.g., local, similar sized institutions with CRPs; well-established, exemplar CRPs, such as Texas Tech that had program development worksheets available). Commonalities across the research literature, fellow institutions, and student recommendations resulted in two initial foci: Mutual support in the form of drop-in peer to peer recovery meetings, and mindfulness/relaxation bi-weekly group sessions. Grounded in a harm reduction approach, and consistent with the All Recovery mutual-self-help meetings described by Vest et al. (2025), the drop-in peer recovery meeting was intended for students in recovery or curious about recovery, and their allies, to connect with and provide mutual support to campus peers. The meeting was held at the same time and place throughout the semester and facilitated by a student with a recovery identity. Similarly, flyers were posted around campus advertising the mindfulness programming; biweekly meetings were scheduled to focus on learning and practicing mindfulness techniques (e.g., deep breathing, muscle relaxation) with a health professions graduate student. Additionally, members of the CRP core development team and task force represented our work at numerous tabling events on campus to enhance visibility and awareness. We strived to be creative in how we involved students who were interested in supporting peers in recovery. For example, one student with interest and experience in community-based participatory research worked with the co-director to develop a cannabis education and harm reduction workshop for collegiate settings, which emerged as a priority area from task force meetings and literature reviews.

#### Component 6: Apply to at least one professional conference or journal outlet to contribute to the scholarly literature on CRPs

Although it is growing, the literature on CRP development, implementation, sustainability, and outcomes is relatively limited [13, 16]. In our efforts to promote CRPs on campuses to support students in recovery, contributing to the professional literature seemed paramount. We presented at two professional organizations’ annual conferences, which resulted in subsequent contact by one insititution looking to start a CRP and one research partnership evaluating a health-focused harm reduction workshop for college students.

## Conclusion

The following recommendations emerge from lessons learned as a CRP co-director in year 1 of launch. Program development, particularly in a space like CRPs where no one “gold standard” model exists and individualizing programming to one’s respective campus culture is key, is a time and resource intensive, fluid process that evolves over time. Creating a network of individuals that represent vital perspectives to collaborate and brainstorm with and reviewing content in the gray and empirical literature to inform your programming (e.g., Texas Tech program replication materials; ARHE resources; scholarly literature emerging from Noel Vest and colleagues) is strongly recommended.

### If you are starting a CRP, be patient with and kind to yourself

We faced disappointments (e.g., we could not get a dedicated space on campus to house our CRP) and challenges (e.g., elongated time to get approval for components of the program). Most activities took more time to acquire than we had initially anticipated. Those of us who do this work are passionate about our allyship, serving the community, and dismantling stigma. This passion can sometimes translate to wanting to do as much as possible as quickly as possible and a willingness to start again from scratch if initial efforts are not overwhelmingly successful. Patience and self-compassion are key. New CRP programs are encouraged to build flexibility into their program timelines to support realistic goal attainment and prevent burnout among CRP staff and contributors.

### Leverage existing resources

Get to know your campus, your community, and your students. Instead of reinventing the wheel, establish partnerships, share resources when possible, and ask your colleagues about lessons learned in program development. Encourage your students to take leadership positions and support their personal and professional development in the process. The CRP community is invested in sharing materials and resources. Further, the ARHE is building a resource compendium on their website. Explore opportunities, look for what you need before you develop it yourself, and outreach the CRP community for resources.

As well, reaching out to established CRP programs for mentorship and support is highly encouraged. These relationships buoyed our efforts in many ways, while encouraging us to adapt our program to our unique campus culture.

### Be mindful of the impact of stigma and misinformation

Stigma continues to pervade the SUD field, and that includes stigma encountered in higher education. The impact of stigma on people living with SUDs or in recovery is underestimated. Throughout your CRP development, reflecting on the ways in which stigma may impact the processes and success of your CRP may yield valuable insights.

In terms of misinformation, for example, one challenge we faced was utilizing harm reduction practices and resultant concerns that this could inadvertently promote student substance use. This encourages the recommendation to be mindful of your own assumptions about others’ knowledge, and commit to creating learning opportunities to share your expertise. It can be easy, in the moment, to experience frustration, and perhaps deflation, but turning that frustration into action to educate others and disseminate evidence-based findings is powerful.

### Self-disclosure in the CRP community

Something that surprised and inspired us was the openness within the community to disclose one’s recovery status—at my first conference, a colleague’s exact words were “the anonymous are out.” I had not anticipated directly being asked my recovery status and the recovery status of other members of the CRP staff. Answering for myself fits squarely within my comfort zone, whereas answering for others does not. Having these interactions encouraged me to prepare my team for potentially being asked those questions, and to make informed decisions about public disclosure (e.g., on your university website).

### Institutional buy-in is necessary, but not sufficient

The initial success of our CRP would not have been possible without institutional support from university administration and campus leadership, combined with financial support through grant funding, and a highly motivated start-up CRP team. Specific to institutional buy-in, campus administrators may fear that having a CRC/P may indicate to the public that substance use is a problem on campus—reviewing the literature that demonstrates how inclusion at the institutional level has been shown to promote outcomes among students with “invisible” identities can help address this concern (e.g. [40]). Developing assessment tools to better understand student needs and risk perceptions associated with substance use on your campus, inviting campus partners to join your efforts, working with students and colleagues to launch initial programming, building relationships with internal and external stakeholders, and organizing events took time, flexibility, and endurance. The program materials in the figures can reduce some of the program development lift as it relates to assessing student needs and recruiting campus colleagues for new CRPs.

### Consider cultural accommodations from the outset

The items we developed to assess substance use and recovery on our campus could have been improved in several ways. First, items related to identity (e.g., race, age, ethnicity, sexual orientation, gender, gender identity) were not included in the survey. Future program developers are encouraged to include such items to better understand intersectionality and to accommodate diversity into CRP programming. If feasible, ascertaining the representativeness of the sample respondents in terms of the entire student population, or at least among those in recovery, would have been helpful in interpreting and utilizing the survey findings.

### Assess student needs consistently and in real time

Student interests and needs change somewhat frequently and unpredictably, making regular assessment and data-driven decisions around where to allocate limited resources based on the most recent assessment information available important. Coupled with the ever-evolving student body, awareness of student interests and flexibility on behalf of programs and administrators to pivot, is clearly needed.

Further, closely reviewing Hennessy et al.’s *Recovery Capital Logic Model for CRP Evaluation* [16], and consulting with several CRP program directors around their methodology for measuring program outcomes and impact, is highly recommended. Although the faculty statistician on our team was ready and willing to run the data, what to measure, when to measure it, and how best to measure it was not clear from the outset and remained elusive throughout the project. This expertise is critically needed when starting a new CRP to demonstrate to decision makers and funders the importance of the work being conducted and the need for the CRP.

### Collaboration is important and individual responsibilities are okay, too

The graduate RAs on our core CRP development team shared project-related responsibilities. In retrospect, charging one RA with project management, overseen by one of the co-directors, and tasking the second RA with community outreach and event planning efforts, overseen by another co-director, would likely have proved more efficient. Especially in year 1, networking is critical, as is an on-campus presence to familiarize the campus community with the CRP and its goals. Simultaneously, ensuring that project goals are completed on time and within budget is also crucial to project success. Having both RAs engage in project management and event planning/outreach activities, and the co-directors overseeing all efforts, led to an uneven distribution of work and periods of high stress at times depending on, e.g., the RA and co-directors’ strengths, availability, and cumulative project activities for the week.

### Engage in self-reflection and performance improvement

Strategically and consistently reflecting on the research literature, local student assessments, and your own observations can reduce potential bias and enhance program effectiveness. Creating space for reflection can seem like a luxury or an inefficient use of time, but we argue to the contrary, as doing so creates opportunity to carefully consider alternate perspectives and engage in creative problem solving.

Building a CRP grounded in student needs and engaging students to participate will undoubtedly take time and be accompanied by numerous setbacks, but as we were repeatedly reminded by colleagues, “If you build it, they will come.” As one colleague shared, CRPs can prove the most valuable resource students in recovery receive during their college careers. Creating opportunities for students to connect with others with similar experiences and to access campus supports may mean the difference between return to substance use or sustained recovery, completing one’s degree or leaving campus early.

## Data Availability

No datasets were generated or analysed during the current study.

## Abbreviations

AOD: Alcohol or other drug use
ARHE: Association of Recovery in Higher Education
CRC: Collegiate Recovery Community
CRP: Collegiate Recovery Program
CRP TF: Collegiate Recovery Program Task Force
NCHA: National College Health Assessment
RA: Research Assistant
SME: Subject Matter Expert
SOP: Standard Operating Procedures
SUD: Substance Use Disorder
